# Potential for Wearable Sensor-Based Field-Deployable Diagnosis and Monitoring of Mild Traumatic Brain Injury: A Scoping Review

**DOI:** 10.3390/s25092803

**Published:** 2025-04-29

**Authors:** Hope C. Davis-Wilson, Erika Maldonado-Rosado, Meghan Hegarty-Craver, Dorota S. Temple

**Affiliations:** RTI International, Research Triangle Park, NC 27709, USAmhegarty@rti.org (M.H.-C.); temple@rti.org (D.S.T.)

**Keywords:** mTBI, inertial sensor, accelerometer, heart rate variability, postural sway, gait

## Abstract

Studies have shown that wearable commercial off-the-shelf sensors, such as accelerometers, inertial measurement units, and heart monitors, can distinguish between individuals with a mild traumatic brain injury (mTBI) and uninjured controls. However, there is no consensus on which metrics derived from wearable sensors are best to use for objective identification of mTBI symptoms. The primary aim of this scoping review was to map the state of knowledge of wearable sensor-based assessments for mTBI, based on previously published research. Data sources included Web of Science and PubMed. Original peer-reviewed articles were selected if mTBI was clinically diagnosed, an uninjured control cohort was included, and data collection used at least one digital sensor worn on the body. After screening 507 articles, 21 studies were included in the analysis. Overall, the studies identified multiple wearables-derived physiological metrics that differ between individuals with mTBI and uninjured controls. Some metrics associated with static balance, walking tasks, and postural changes to initiate an autonomic nervous system response were shown to support diagnosis of mTBI in retrospective studies with acceptable to outstanding accuracy. Further studies are needed to formulate standard protocols, reproduce results in large heterogeneous cohorts in prospective studies, and develop improved models that can diagnose mTBI with sufficient sensitivity and specificity in targeted populations.

## 1. Introduction

Mild traumatic brain injury (mTBI), commonly referred to as a concussion, is a highly prevalent injury in sports and military settings [1,2]. Between 3 and 4 million mTBI cases are reported annually in the United States, with a large portion of these the result of sports [2]. Additionally, mTBI is known as the “signature injury” in Veterans who were deployed during wars in Iraq and Afghanistan; 8–20% of these Veterans received an mTBI diagnosis during service because of repetitive blunt force or blast exposure [1]. Sustaining mTBI can have lasting impacts on long-term health, including increased risk of cardiovascular disease [3], epilepsy [4], and mental health conditions [5]. In military deployment, where it is difficult to immediately see a clinician, mTBI cases may go undiagnosed. Individuals with undiagnosed mTBI may have more severe post-mTBI symptoms compared to individuals who receive an early diagnosis [6].

At present, there are no objective, gold standard measures to diagnose mTBI. The Sideline Concussion Assessment Tool (SCAT) and the Military Assessment of Concussion Evaluation (MACE) are often used to diagnose mTBI in the field in sports and military settings, respectively [7,8]. Both rely on a series of quick tests that include a symptom checklist, postural balance assessment, and cognitive evaluation. Similar tests, but more thorough and augmented with general-purpose imaging and blood tests, are conducted in clinical settings [9]. Although the diagnostic approaches are considered moderately accurate, the methodology is largely subjective and therefore vulnerable to inconsistency and lack of sensitivity in people with less severe symptoms [10]. Moreover, neurocognitive symptoms can be subtle and vary among individuals as to their magnitude and evolution over time [11]. Reliance on patient recall and subjective reporting of symptoms, which may be influenced by the desire to return to play or duty more quickly, can introduce bias in mTBI diagnoses [11]. Objective, field-deployable methods that quickly and reliably diagnose mTBI following blunt force or blast exposure are needed to improve health outcomes following mTBI.

Great efforts are being made to identify objective imaging [12,13], biochemical including blood-based [14,15,16], and other digital outcomes [17,18,19,20] related to mTBI. Of these outcomes, digital biomarkers derived from wearable sensors, or wearables, offer the promise of a low-cost, non-invasive method to diagnose mTBI and monitor the injured individual’s recovery, especially outside of clinical settings. Commercial off-the-shelf wearables are being developed at a rapid pace; they are able to collect high-resolution digital data that can be used to objectively detect changes in physiological outcomes. For example, wearable electrocardiogram (ECG) sensors can detect changes in autonomic nervous system (ANS) functioning by measuring heart rate variability (HRV) [21], and wearable accelerometer and inertial measurement unit (IMU) sensors can detect subtle changes in balance [19] and gait [20].

Studies have demonstrated that the wearables can discern between individuals with mTBI and uninjured controls. However, there is no consensus on the methodology because of the wide range of sensors being used, outcomes being measured, and tasks being performed to test the mTBI-related symptom (i.e., changes in posture to test ANS response, gait tasks, or more challenging movements to test the vestibular system). This review seeks to map existing literature with regard to the use of wearable sensors to diagnose or monitor mTBI. We define a wearable sensor as a device that transmits digital biometric signals in an autonomously operating, self-contained, and wireless unit [22]. We focus on wearable devices that can be easily deployed outside of clinical settings and are readily available in the commercial market; specifically, accelerometers, IMUs, and heart rate monitoring sensors.

The research questions for this scoping review are: (1) What approaches have been explored to diagnose or monitor mTBI using wearable sensors? and (2) What is known about accuracy of the wearables-based techniques? To answer the questions, we identify the protocols and analytical methods used in previously reported research and assess whether the methods are similar enough to allow for comparison among the studies. Results of the review provide an overall picture of the current state of the field and thus help identify knowledge gaps in the application of wearables to the diagnosis and monitoring of mTBI. The results will inform future systematic reviews by identifying study designs that have the potential to support focused research questions concerning healthcare practice.

## 2. Materials and Methods

### 2.1. Search Strategy

We used the scoping review guidelines developed by Joanna Briggs Institute [23] and a written protocol to conduct an exhaustive electronic search of the Web of Science and PubMed databases. A reference librarian at RTI International assisted the investigators in performing the search. The following search was performed initially on 29 September 2022 and updated on 16 December 2024: (“mTBI” OR “concussion” OR “mild traumatic brain injury” OR “traumatic brain injury” OR “sub-acute head injury”) AND (“wearable” AND “sensor”) OR “IMU” OR “heart rate variability” OR “accelerometer” OR “autonomic nervous system” OR “heart rate metric” AND “cardiovascular response”. We included studies published since 1 January 1960. Bibliographies from relevant studies found in the initial online search were cross-referenced to identify additional relevant articles.

### 2.2. Inclusion and Exclusion Criteria

A wearable sensor-based assessment for mTBI included any protocol performed in a laboratory or clinical setting that used data from at least one wearable sensor for the diagnosis or assessment of mTBI in human participants compared to uninjured human control participants. Two reviewers (H.D.-W., E.M.-R.) identified articles as eligible if they met the following criteria based on our previously defined clinical question: (1) enrolled human participants with mTBI diagnosed by a physician, (2) reported data collection using at least one digital sensor worn on the body, and (3) inclusion of a control cohort. Articles were excluded if they involved (1) mTBI treatment used to alter physiological outcomes other than standard-of-care treatments, (2) control participants who had a previous history of mTBI within 6 months of the study, (3) self-reported mTBI diagnosis (i.e., no clinician diagnosis and only self-reported symptoms), and (4) did not provide specific objective outcomes from the wearable. Only articles written in English were considered for review, and abstracts, case studies, conference proceedings, reviews, commentaries, discussion papers, or editorials were excluded.

### 2.3. Data Extraction

Data were extracted by the primary reviewer (HDW) then synthesized into table format, with a second reviewer (ER) confirming the data. The following data were extracted from each article included in this systematic review: title, authors, journal of publication, year of publication, sample size, subject characteristics, time since mTBI event, study design, experimental setting, experimental protocol, instrumentation, statistical analysis, and wearable sensor outcomes and results. Some studies included receiver operating characteristic (ROC) curves, providing information on sensitivity and specificity of diagnostic methods [24], as part of their statistical analysis. Area under the ROC curve, AUROC, is used as a measure of accuracy of the diagnosis. In general, an AUROC of 0.5 suggests no discrimination, 0.7 to 0.8 is considered acceptable, 0.8 to 0.9 is considered excellent, and more than 0.9 is considered outstanding.

## 3. Results

### 3.1. Selected Studies

**Figure 1:**
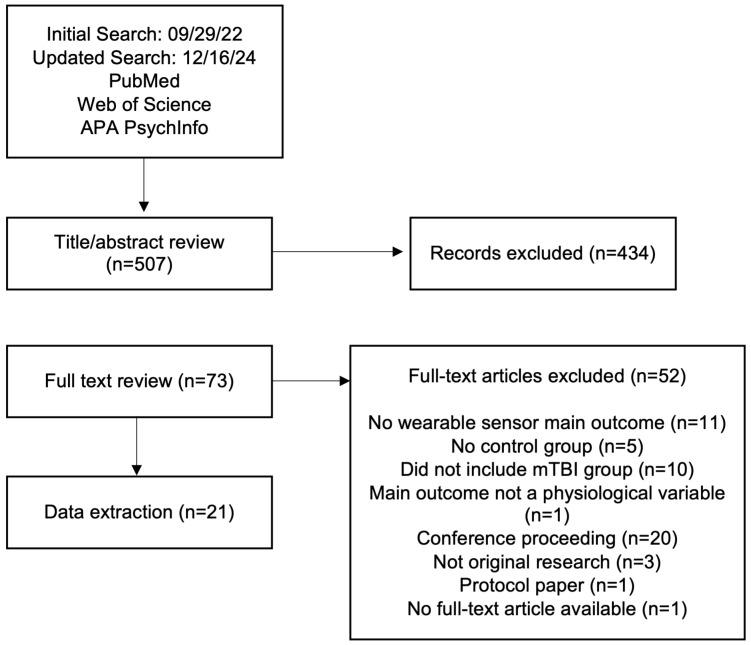
Flow diagram of the search protocol.

A total of 507 unique records were identified from our database search. After screening the abstract and title for keywords, 73 full-text articles were evaluated further for eligibility and 52 articles were excluded (Figure 1). Figure 1 describes why full-text articles were excluded from the analysis. Our analysis of the remaining 21 papers led us to categorize them by the type of task used to discern between mTBI and uninjured controls. The three categories are: (1) static balance tests (SBT), (2) walking tasks (gait tests, GT), and (3) postural changes to initiate an ANS response (heart rate variability tests; HRVT). The articles are listed alphabetically by the first author in Table 1, along with the information on wearable sensors used in the studies and demographics of study participants.

### 3.2. Static Balance

#### 3.2.1. Study Participants

Studies recruited adults and adolescents from university settings [17,25,30], university-affiliated emergency departments [27,28], university-affiliated sports medicine clinics [18], and local clinical sites [36]. One study recruited adults within the Department of Veterans Affairs health care system [33].

#### 3.2.2. Wearable Sensors and Protocols

A single IMU sensor placed on the sacrum was used in four of the studies (83%) [17,18,30,33]. In other studies, three Shimmer IMU sensors were placed on the sacrum and anterior portion of each leg-shank [27,28], two IMU sensors were placed on the forehead and sternum [25], and one IMU sensor was placed behind the ear [36].

While wearing the sensors, participants in three of the studies completed the Balance Error Scoring System (BESS) or modified BESS (mBESS) tests [17,18,27], which are frequently used clinical tools for post-mTBI postural control assessment [41]. In the BESS test, participants perform a series of 20-s stances on a firm surface: double-leg stance, single-leg stance, and tandem stance on both firm and foam surfaces; the mBESS utilizes only firm surfaces. Clinical uninstrumented BESS assessments require a clinician to count the number of errors from proper stance that occur throughout the 20-s trial (i.e., opening eyes, lifting hands off of iliac crest, stepping to regain balance, stumbling or falling out of position, abducting or flexing the hip by more than 30 degrees). IMU sensors, on the other hand, provide an objective measure of the postural sway; the instrumented BESS or mBESS test can therefore be used in the field even in the absence of a clinician. The same applies to the instrumented modified clinical test of sensory integration for balance (mCTSIB) [30,33] or similar balance [25,36] or postural adjustment tasks [28].

#### 3.2.3. Measurement Outcomes

**Figure 2:**
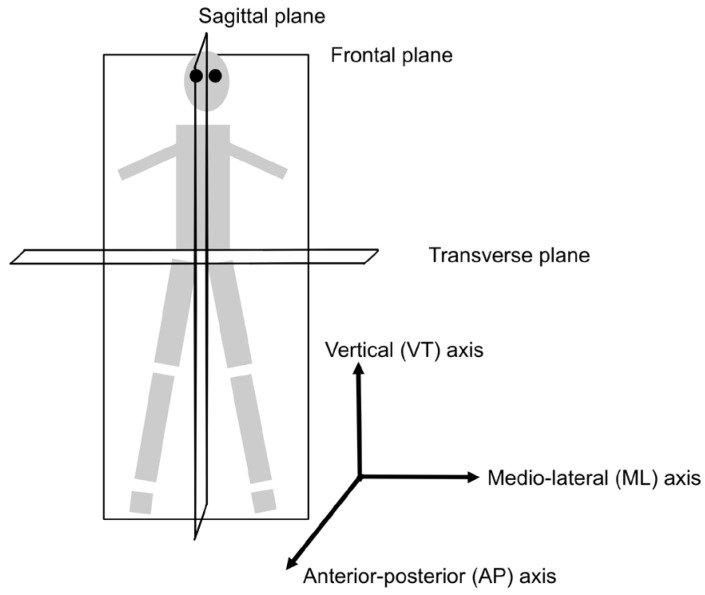
Axes of rotation and anatomical planes. Anterior-posterior (AP) axis runs front to back, medio-lateral (ML) axis runs side to side, and the vertical (VT) axis runs top to bottom through the pivot point. Reprinted from [42] under CreativeCommons license.

All eight studies employing static balance tests (100%) were cross-sectional [17,18,25,27,28,30,33,36]. Table 2 lists specific measurement outcomes investigated in each of the studies. Postural sway calculations from IMU-collected data varied between studies, but each method utilized the anteroposterior (AP), mediolateral (ML), or vertical (VT) acceleration time series to calculate the displacement and acceleration of the center of mass (COM), see Figure 2.

Specifically, studies calculated postural sway using the root mean square (RMS) of the acceleration time series [17,18], the average power of the acceleration time series [36], or the area/volume of sway from the COM [25,27,28,30,33]. One study [18] showed that the instrumented BESS (AUROC = 0.70 [0.50–0.91]) and mBESS (AUROC = 0.81 [0.64–0.99]) provided acceptable to excellent diagnostic accuracy for differentiating mTBI from control participants, 2 to 13 months after the injury (Table 2). Average power collected by an IMU sensor placed behind the ear during eyes-closed and eyes-open balance tasks within 30 days after the injury predicted mTBI diagnosis with outstanding accuracy (AUROC = 0.98 [0.96–0.99]). Other studies did not provide ROCs for mTBI diagnosis, although Doherty et al. [27] and King et al. [17] constructed ROC curves for detecting whether the individual succeeded or failed in an instrumented balance test, using the determination by a human observer as a reference. The capability to count errors in balance tests, based on IMU-provided signals, can provide objective, accurate BESS-scores without the need for a clinician.

Two studies of postural sway evaluated sensory reweighting, or the ability to integrate sensory information from the visual, vestibular, and proprioceptive systems using the mCTSIB [30,33]. The IMU sensor provided a ratio of postural sway during eyes-open and eyes-closed conditions. Sensory reweighting was calculated by comparing postural sway between eyes-closed and eyes-open static balance conditions; a score of 1.00 indicated no difference between eyes-closed and eyes-open conditions, and a higher score indicated that an individual relied more on vision for balance. When standing on foam, participants increased their postural sway more than the control group under eyes-closed conditions 2–3 days following mTBI [30]. Similarly, postural sway averaged across all conditions of the instrumented mCTSIB test discriminated between participants with an mTBI and persistent balance complaints (>3 months post-mTBI) and the uninjured control group with acceptable accuracy (AUROC = 0.77 [0.60–0.75]) [33].

One study evaluated angular velocity and range of motion in participants recovering from mTBI (months after the injury) and found slower angular velocity, both forehead and sternum, and greater range of motion when compared with controls [25].

### 3.3. Gait Tests

#### 3.3.1. Study Participants

In our review, eight articles [19,20,25,29,32,34,35,40] evaluated walking gait and one evaluated free-living physical activity (primarily walking activity) [38] with wearables for individuals with mTBI and uninjured controls who wore wearable sensors. Studies recruited service members from a military base [29], collegiate athletes in a university setting [19], people from community settings [20,25,32,35,40], children from a children’s hospital [38], and local health clinics [34].

#### 3.3.2. Wearable Sensors and Protocols

All studies utilized IMU or accelerometer sensors to collect gait and physical activity outcomes. Sensor placement and number varied, with studies ranging from one sensor on the waist [35,38,40] or sacrum [20]; two sensors placed on the forehead and sacrum [25,29]; three sensors placed on the lateral ankles and back [34]; four sensors placed on the forehead, sacrum, and each anterior tibia [19]; and seven sensors placed on the forehead, sternum, waist, wrists, and feet [32]. Three studies utilized a cross-sectional laboratory design [25,29,32], and three were longitudinal laboratory designs, spanning from initial assessment within 72 h to 2 months post-mTBI [19,20,34]. Three studies assessed free-living gait and physical activity outside of the laboratory over a 7-day period [35,40] and 4-week period [38].

#### 3.3.3. Measurement Outcomes

Table 3 lists those measurement outcomes that were statistically different between the mTBI and control groups. Outcomes included duration/time, length, angle, linear velocity, angular velocity, and acceleration, all derived from triaxial accelerometer time-series signals from the IMU sensor. Sharma et al. [38] reported physical activity outcomes from an accelerometer including sedentary time, light physical activity, moderate physical activity, and vigorous physical activity time.

Participants with mTBI demonstrated significantly slower peak head angular velocity when rotating their head while walking, 2 and 5 days post-mTBI [19]. The same effect was observed in participants recovering from mTBI months after the injury [25,32]. Peak head angular velocity during gait tested 2 days post-mTBI had acceptable diagnostic accuracy (AUROC = 0.73 [0.56–0.85]) when differentiating people with mTBI and uninjured controls (Table 3) [19]. Larger increases in peak head angular velocity over time (from 2 to 10 days) were associated with greater total symptom severity [19].

Two studies evaluated dual-task gait (walking while performing the Stroop test) and found that people with mTBI walked slower than uninjured controls at 3 days, 1 week, 2 weeks, and 2 months post-mTBI [20,34]. ML peak accelerations during the second half of the gait cycle (double support and swing phase) were lower in the mTBI group through 2 months post-mTBI compared to uninjured controls. The ROC curves based on data obtained within 72 h and then 1 week post-mTBI demonstrated excellent accuracy at distinguishing between mTBI and uninjured groups, with AUROC = 0.89 and 0.81, respectively [20]. Pitt et al. found that participants diagnosed with mTBI demonstrated slower angular and roll velocity during loading and mid-stance compared to uninjured controls [34].

No significant differences were found in free-living spatiotemporal gait metrics (stride time, stance time, step width, etc.) in individuals with an mTBI compared to uninjured controls [35]. Individuals with chronic mTBI symptoms demonstrated impaired turning characteristics under free-living conditions. Specifically, individuals took longer to turn and demonstrated lower turning velocity compared to uninjured controls [40]. No differences were found between daily steps or physical activity duration between groups [40]. Differently, children with acute mTBI spent more time being sedentary and less time being physically active compared to controls [38]. Favorov et al. [29] tested participants with a military-relevant tactical agility assessment involving rapid movement transitions while running and carrying a simulated weapon. Phases of (1) lowering, (2) rolling, and (3) rising and running were evaluated. Participants with an mTBI performed each phase of the test slower compared to healthy controls, and combined duration of lowering and rolling phases was the most accurate differentiator of mTBI from control participants (AUROC = 0.83 [0.72–0.93], Table 3). Later trials show more of a difference between the groups and indicate that the lowering phase is the most sensitive through all trials [29].

### 3.4. Heart Rate Variability Tests

Previous laboratory-based studies have demonstrated changes in HRV post-mTBI using 12-lead ECG measurements [42]. We identified several studies that used HRV measured by wearable sensors to distinguish between injured individuals and controls.

#### 3.4.1. Study Participants

The reviewed studies recruited athletes [26,31,37,39]. One study involved active military personnel within 72 h of a medically diagnosed mTBI [21].

#### 3.4.2. Wearable Sensors and Protocols

Two studies utilized sensors based on photoplethysmography (wrist-worn Empatica E4 [26] and finger-worn PPG sensor [37]). Three studies utilized ECG sensors (Zephyr BioHarness strap with BioModule sensor [31], Bittium Faros 180 [21], and AD Instruments LifeMonitor [39]).

**Figure 3:**
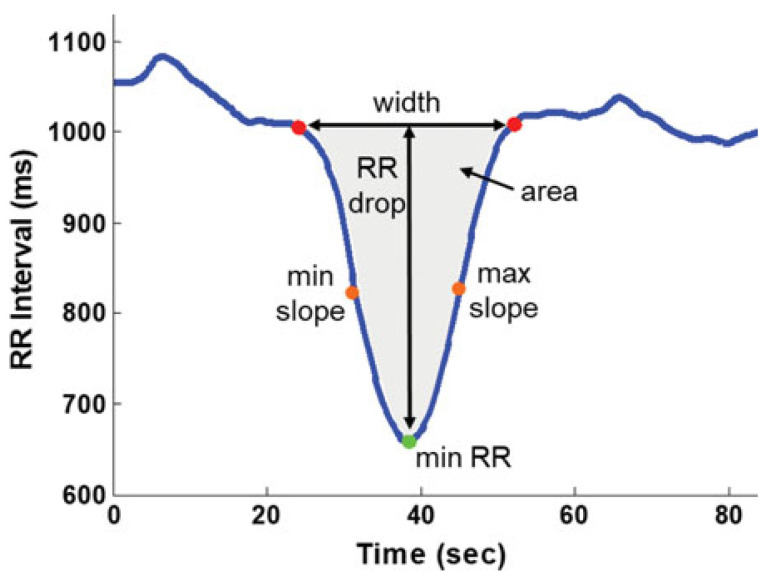
Illustration of parameters calculated in the transition period from lying to standing and sitting to standing. Reprinted with permission from [21].

One study monitored HRV during sleep over a period of the individual’s medically specified recovery and 3 weeks after return to sport [26], another during daytime recording sessions conducted months to years after the injury [31]. One study monitored HRV following an exertion test in a clinic for the return-to-sport decision [39]. Two studies monitored HRV during lie-to-stand transition [21,37] and sit-to-stand transition [21]. The study by Sas et al. evaluated participants at a single time point during the acute postinjury phase (<30 days) [37]. In addition to HRV, the study by Russell et al. monitored parameters of the change in the interbeat interval during the transition as illustrated in Figure 3.

#### 3.4.3. Measurement Outcomes

Table 4 lists outcomes for the studies that used HRV to distinguish between mTBI and control groups.

As illustrated in Table 4, no statistically significant differences in nocturnal HRV were observed between the two cohorts [26]. Similar outcome was observed in another study that compared HRV metrics during daytime recording sessions conducted months to years after the injury; however, differences in the HRV characteristics were evident after a cognitive test or aerobic exercise [31]. No differences in HRV characteristics were observed following a dynamic exertion test conducted to assess readiness to return to sport several weeks after the injury [39].

Two studies recorded HRV metrics during postural changes [21,37]. Whereas both studies saw reduced HR during the lie-to-stand transition, only the first study was powered sufficiently to differentiate between mTBI and control groups [37]. A model using the chair-to-stand transition and the lie-to-stand transition HRV metrics achieved excellent diagnostic accuracy (AUROC = 0.88) for differentiating between mTBI and uninjured controls; however, AUROC CI was not calculated for this small (n = 31) cohort [21].

## 4. Discussion

### 4.1. General Observations

This scoping review evaluated literature that reported using wearables to identify differences between individuals with an mTBI and uninjured controls for either diagnosing mTBI or monitoring symptoms of the injury over time. Although the search for blood-based biomarkers continues with some success, as evidenced by the recent approval of a test utilizing GFAP and UHCL1 proteins in blood to help rule out the need for head and brain imaging [14], emerging wearables offer an opportunity to provide non-invasive diagnostic and monitoring tools that can be implemented outside of clinical settings. Although previous literature has shown that balance [41,43], gait [44], and HRV [45] outcomes collected in laboratory conditions are sensitive to change following mTBI, our review is the first to our knowledge that identified studies that used wearables to discern between individuals with an mTBI and uninjured controls in testing protocols that could be administered in the field. Wearables show promise to be utilized in free-living settings because of their low cost and ease of use.

The phased approach to the development of a diagnostic test used by Leeflang et al. [46] provides a convenient framework for assessing the maturity of methodologies we reviewed. All of the studies in this review identified metrics that effectively discriminated between individuals with and without mTBI in laboratory conditions. Several of the studies advanced beyond Phase 1 by developing algorithms that integrated multiple metrics extracted from the wearables data. These Phase 2 investigations resulted in the determination of the sensitivity and specificity of the proposed algorithms and the construction of ROC curves. Although we have not identified any Phase 3 studies—prospective in design and conducted in real-world settings—the Phase 1 and 2 papers we reviewed provide a strong foundation for future research in this area.

### 4.2. Static Balance

Eight studies assessed instrumented balance tests for identifying acute and chronic mTBI patients. Detecting balance and gait deficits in acute and chronic mTBI patients is difficult because clinical assessments typically rely on observational analysis. This subjective scoring is less reliable than objective measures, particularly when evaluating chronic mTBI symptoms over a longer period [47,48]. In the studies using wearable sensors, the BESS test and the mCTSIB were utilized to quantify postural sway and sensory reweighting without the need for a clinician. Results from one of the studies suggest that evaluating postural sway (via one accelerometer or IMU sensor placed on the sacrum) during a clinical balance assessment (BESS, mBESS, or mCTSIB) will classify individuals with mTBI versus uninjured controls with fair to excellent accuracy (AUROC range: 0.70–0.81) [17,18,27,28]. In another study, Ralston et al. found that average power (measured from an accelerometer behind the ear) across two balance conditions (eyes open and eyes closed on a firm surface) provided outstanding diagnostic accuracy between mTBI and control groups (AUROC = 0.98 [0.96–0.99]). Although Ralston et al. [36] demonstrated the highest mTBI AUROC for static balance tests, there were differences in participant population between studies that prevent making a direct comparison. For example, Ralston et al. recruited individuals who had an mTBI event in the past 30 days, while other studies evaluated individuals who had an mTBI within the past 72 h.

The studies demonstrate that IMU sensors can provide objective and sensitive measures of static balance; however, further work is needed to develop standard protocols and procedures for extracting the biomarkers from wearables data; replicate study results in larger, more heterogeneous cohorts that allow for the investigation of endogenous and exogenous confounding effects; and determine the sensitivity and specificity of the methodology in prospective studies in real-life situations.

### 4.3. Gait and Dynamic Tasks

Although static balance is an important component of mTBI rehabilitation, static balance tests do not capture more dynamic movements that are required for daily activities and sports and should be monitored during recovery. Peak ML acceleration during dual-task gait demonstrated excellent diagnostic accuracy 72 h (AUROC = 0.89) and 2 weeks (AUROC = 0.81) post-mTBI [20]. However, CI ranges for the AUROC values were not given and are likely to be significant considering the small sample size, n = 10. Longitudinal studies found that gait deficits were no longer distinguishable between participants with mTBI and uninjured controls beyond 2 weeks post-mTBI except for gait speed, which remained slower in the mTBI through 2 months post-mTBI [20].

Cross-sectional studies found that individuals post-mTBI performed more complex movements (turning during gait, lowering from standing to prone) more slowly compared to uninjured controls regardless of time post-mTBI [29]. Results suggest that evaluating movement duration during complex movement tasks (dual-task gait, transition time while lowering or rising to/from the ground, turning during gait) may offer the most clinical utility considering that these deficits persist months post-mTBI and time duration is typically a metric that is easy to calculate in a clinical setting. One limitation of these gait and dynamic tasks is that the methods were different in different papers. The lack of consensus on methods to evaluate gait using wearables post-mTBI highlights the need for replication of study results in larger populations and various timepoints post-mTBI.

### 4.4. Heart Rate Variability

Exaggerated sympathetic nervous system activity has been reported post-mTBI [49,50]; however, cardiovascular response following mTBI has been underreported in humans. Two recent studies evaluated resting HRV metrics during sleep or daytime using wearable ECG sensors. Both found no significant differences in the metrics between individuals with mTBI and uninjured controls, suggesting limited diagnostic value of resting HRV values. One study evaluated resting HRV prior to and immediately following an aerobic exercise protocol used to determine return to sport/activity [39]. Participants with an mTBI demonstrated lower SD of successive heartbeats and root-mean-square of differences between successive heartbeats following the exercise protocol compared to controls [39]. One study in this review evaluated ANS function during postural changes [21]. Russell et al. found that HRV measured using a wearable ECG monitor can be used to distinguish between individuals with mTBI and uninjured controls [21]. Specifically, individuals with mTBI take longer to reach steady HRV following a postural change compared to uninjured controls. Few studies have evaluated ANS dysregulation and cardiovascular function following mTBI outside of a laboratory setting, but there is a significant need to further understand subsequent cardiovascular effects following mTBI [3]. Results from this review conclude that HRV may be a promising metric derived from a wearable sensor for mTBI detection because it is free from the influence of participant effort, and the technology to measure HRV is clinically accessible and field deployable. This review highlights the need for more studies to utilize wearable ECG methods to detect mTBI.

### 4.5. Methodological Limitations and Confounders

This review identified several gaps in the literature for using wearable technology to diagnose and monitor recovery following mTBI. All studies included a control cohort; however, the definition of uninjured control varied. Uninjured controls were most often defined as having no self-reported history of mTBI within the last 6 months [17,19,30] or within the last year [20,21,27,28,29,34]. Studies were also inconsistent with the age of participants. Two studies included only adolescents [31,38], six studies included children and adults [20,27,28,36,37,39,51], and 12 studies included only adults [17,18,19,21,25,29,30,32,33,34,35,40]. One study did not specify age range [26]. Many studies had wide age ranges and included both young adults (~18 years) and older adults (45–60 years) within the same study [25,29,32,33,35,36,40]. Age influences HRV [52] and postural balance [53], therefore a wide age range may confound results in studies included in this review. Four studies looked at active military or Veteran populations [21,29,33,35]. It is likely that the mechanism of injury was different between civilian and military populations; however, few studies indicated mechanism of injury. Future work should include information regarding mechanism of injury. Most studies included in this review were cross-sectional [17,18,21,25,27,28,29,30,31,33,35,36,37,39,40] and evaluated participants within days to months following mTBI. Future studies should consider evaluating participants at multiple time points starting within 3 days of mTBI to capture symptoms at the time of the mTBI and change in symptoms as an individual recovers. Postural sway was measured using a variety of techniques between studies [17,18,27,28,30,33]. Nine of the 21 studies included AUROC values, but only seven of these studies included 95% CIs of the AUROC. Studies should report 95% CIs to allow for comparison between studies with different sample sizes. Consensus on measurements and statistical analyses will be needed prior to widespread clinical use.

Potential confounders exist for the described mTBI assessment methods. For example, individuals with mental disorders, such as anxiety, depression and schizophrenia, may show deficits in balance, gait, and posture [54]. For populations such as athletes or active military personnel, creating the individual’s baseline of metrics of interest prior to the potential exposure to conditions that may result in mTBI may be practical and could minimize the confounding bias.

Studies in this review focused on using accelerometers, IMUs, and ECG sensors to evaluate postural sway, gait, and HRV. Wearable sensor development is ever evolving, and as new sensors enter the market, they may be useful in diagnosing and monitoring recovery of mTBI. The current study did not focus on all field-based technologies such as portable electroencephalography (EEG) systems, because the form factor of currently available EEG wearables is not conducive to longitudinal monitoring in free-living conditions. However, emerging products offer low-profile wireless EEG systems that may ultimately assist with mTBI diagnosis in both field and clinical settings [https://www.bitbrain.com/neurotechnology-products (accessed on 28 April 2025)]. Additionally, there is a need to identify whether combinations of sensors can better detect mTBI, for example combining postural sway, gait, or HRV outcomes. This review demonstrates that clinically feasible wearable technology is available and has the potential to be used to diagnose mTBI and identify balance, gait, and HRV deficits. Finally, this review highlights the limitations of current research including (1) various ages and other confounders, (2) inconsistent documentation of mechanisms of injury, and (3) differences in metric calculations.

## 5. Conclusions

Tests that measure greater postural sway, slower gait tasks, and prolonged ANS response to postural changes provide diagnostic value for mTBI. These field-deployable, non-invasive protocols show promise for providing objective diagnosis and identifying subtle symptoms during recovery for individuals with mTBI. The wearables-based techniques are still in an early stage of development, with only a subset providing sensitivity and specificity metrics and ROC curves. Further studies are needed to evaluate the effect of endogenous and exogenous confounders. Once robust algorithms are established in laboratory conditions, the research needs to incorporate studies that are prospective in design and conducted in real-world settings.

## Figures and Tables

**Table 1:** Information on wearable sensors and demographics of participants in the included studies.

Author, Year	Paper Group	Wearable Sensor (# of Sensors Used)	Participants with mTBI				Control Participants		
			Age (Years) Mean (SD)	Time Since mTBI Mean (SD or Range)	N	% Female	Age (Years) Mean (SD)	N	% Female
Campbell 2023 [25]	SBT, GT	IMU (2)	37 (12)	74 (32) days	73	na	41 (12)	50	Not available
Deling 2023 [26]	HRT	PPG (1)	23 (5)	13 (21) days	18	17 (n = 3)	23 (5)	18	17 (n = 3)
Doherty 2017 [27]	SBT	IMU (3)	22 (4)	9 (7) days	15	27 (n = 4)	22 (4)	15	27 (n = 4)
Doherty 2017 [28]	SBT	IMU (3)	22 (4)	9 (7) days	15	27 (n = 4)	22 (4)	15	27 (n = 4)
Favorov 2021 [29]	GT	IMU (2)	29 (6)	Within 2 years	42	na	30 (6.7)	57	na
Fino 2019 [19]	GT	IMU (4)	20.3 (1.3)	2 (0.6) days	24	25 (n = 6)	20.9 (1.4)	25	24 (n = 6)
Gera 2018 [30]	SBT	IMU (1)	20.6 (1.3)	2–3 days	38	34 (n = 13)	21.0 (1.4)	81	46 (n = 37)
Harrison 2022 [31]	HRT	ECG (1)	16.06 (0.73)	24.13 (17.7) months	16	0 (n = 0)	15.98 (0.62)	18	0 (n = 0)
Howell 2015 [20]	GT	IMU (1)	19.0 (5.5)	2.0 (0.8) days	10	30 (n = 3)	20.0 (4.5)	7	57 (n = 4)
King 2014 [18]	SBT	IMU (1)	16.3 (2.0)	5 (3.3) months	13	77 (n = 10)	16.7 (2.0)	13	77 (n = 10)
King 2017 [17]	SBT	IMU (1)	20.4 (1.3)	2.2 (1.2) days	52	33 (n = 17)	20.6 (1.4)	76	50 (n = 38)
Loyd 2023 [32]	GT	IMU (7)	33.0 (9.5)	244 (21–989) days	45	80% (n = 36)	31.5 (9.5)	46	72 (n = 33)
Martini 2022 [33]	SBT	IMU (1)	39.8 (11.5)	2.3 (2.0) years	41	71 (n = 29)	36.5 (12.1)	53	60 (n = 32)
Pitt 2020 [34]	GT	IMU (3)	20.1 (1.3)	1.8 (0.6) days	11	64 (n = 7)	20.6 (1.9)	11	64 (n = 7)
Powell 2022 [35]	GT	IMU (1)	40.9 (11.8)	440.7 (700.6) days	32	81 (n = 26)	48.6 (22.6)	23	74 (n = 17)
Ralston 2020 [36]	SBT	IMU (1)	18.8 (13.2)	Within 30 days	92	55 (n = 51)	17.2 (7.7)	83	52 (n = 43)
Russell 2020 [21]	HRT	ECG (1)	23.8 (4.6)	Within 72 h	31	10 (n = 3)	24.0 (4.8)	32	12.5 (n = 4)
Sas 2024 [37]	HRT	PPG (1)	15.3	Within 30 days	133	45.9	15.7	100	54
Sharma 2024 [38]	GT	ACC (1)	12.7 (2.8)	Within 4 weeks	60	52 (n = 31)	12.4 (2.7)	60	52 (n = 31)
Sinnott 2023 [39]	HRT	ECG (1)	16.3 (2.3)	18.5 (12.3)	13	31 (n = 4)	16.3 (2.3)	13	31 (n = 4)
Stuart 2020 [40]	GT	IMU (1)	40.2 (12.1)	419 days	29	79 (n = 23)	48.6 (23.1)	23	74 (n = 17)

SBT = Static Balance Tests; GT = Gait Tests; IMU = inertial measurement unit; HRT = Heart Rate Tests; ECG = Electrocardiogram, PPG = Photoplethysmography; ACC = Accelerometer.

**Table 2:** Summary of results for static balance tests.

Task	Condition	Postural Sway Outcome Stratified by Time Since mTBI		
		Days	Weeks	Months
Postural adjustment during gait initiation	Leading with dominant and non-dominant limb		Reduced COM acceleration and displacement [28]	
BESS or Modified BESS	Bilateral stance	Greater COM acceleration, greater power, greater sway area [17]	Greater COM sway volume [27]	
	Tandem stance		No change in COM sway volume [27]	
	Unilateral stance		No change in COM sway volume [27]	
	Average of three stances			Greater COM acceleration; BESS AUROC = 0.70 [0.50–0.91]; modified BESS AUROC = 0.81 (0.64–0.99) [18]
CTSIB	Eyes open, firm surface		Greater COM sway area [30]	Greater COM acceleration [33]
	Eyes closed firm surface		Greater COM sway area [30]	Greater COM sway area [33]
	Eyes closed foam surface			
	Eyes open foam surface			
	Average of four conditions			Greater COM sway area; AUROC = 0.77 (0.60–0.85) [33]
	Sensory reweighting firm surface		No change in COM sway area [30]	
	Sensory reweighting foam surface		Greater COM sway area [30]	
Eyes closed + eyes open balance	Average of eyes closed and eyes open on firm surface		Greater average power, AUROC = 0.98 (0.96–0.99) [36]	
Balance with head turns	Eyes open, firm surface			Slower forehead and sternum peak angular velocity, larger forehead and sternum range of motion [25]

COM = center of mass. BESS = Balance Error Scoring System. CTSIB = Clinical Test of Sensory Interaction and Balance. Results from each study were condensed based on time since mTBI. In cases where a receiver operating characteristic (ROC) was constructed for the purpose of differentiating mTBI subjects from control subjects, AUROC values are given together with 95% confidence intervals (CI) if available.

**Table 3:** Summary of results for gait tests.

Task	Condition	Gait Outcomes Stratified by Time Since mTBI		
		Days	Weeks	Months
Walking	Head rotation	Reduced angular velocity, AUROC = 0.73 [0.56–0.85] [19]		Reduced forehead peak angular velocity [25] Slower and smaller head rotations horizontally and slower head rotations vertically [32] Slower gait speed and greater percent reduction in gait speed during walking with horizontal head rotations [32]
Dual task walking gait	Gait	Reduced speed [20]	Reduced speed [20,34]	NC [20]
	Second half of gait cycle	Reduced peak frontal acceleration [20]	Reduced peak frontal acceleration [20]	
		Reduced peak ML acceleration, AUROC = 0.889 [20]	Reduced peak ML acceleration, AUROC = 0.810 [20]	
	Right heel strike	Reduced VT peak angular velocity [34]	Reduced VT peak angular velocity [34]	
Free-living gait	Turning			Greater number of turns, turn angle and CV, turn duration and CV, peak velocity CV, average velocity CV [40] Reduced peak velocity and average velocity [40]
Free-living PA	Sedentary activity		More sedentary time [38]	
	Light PA		Less light PA time [38]	
	Moderate PA		Less moderate PA time [38]	
	Vigorous PA		Less vigorous PA time [38]	
Tactical agility assessment	Lowering			Greater duration [29]
	Rolling			
	Rising/Running			
	Lowering and rolling			Greater duration, AUROC = 0.83 [0.72–0.93] [29]

ML = Mediolateral; VT = Vertical; CV = Coefficient of variation. In cases where a receiver operating characteristic (ROC) was constructed for the purpose of differentiating mTBI subjects from control subjects, AUROC values are given together with 95% confidence intervals (CI) if available.

**Table 4:** Summary of results of HRV tests.

Task/ Condition	Outcome	Change Stratified by Time Since mTBI		
		Days	Weeks	Months
Sleep	HR, RMSDD, HR CV, RMSDD CV	NC [26]	NC [26]	NC [26]
Awake	Mean RR, RMSDD, SDNN			NC [31]
Post-exertion	RMSDD, SDNN		NC [39]	Greater [31]
Lie-to-Stand Transition	ΔHR	Reduced [37]		
	ΔHR, ΔRSA, ΔLF	NC [21]		
	RR drop, time of max slope, width and area of the valley	Greater [21]		
Sit-to-Stand Transition	Time of min RR, RR drop, time of max slope, width and area of the valley	Greater [21]		

HR = Heart Rate; RR = interbeat interval; RMSDD = root mean square of successive interbeat interval differences; CV = coefficient of variation NC = no change; SDNN = standard deviation of interbeat intervals; Δ = difference between values when standing and lying; RSA = respiratory sinus arrythmia; LF = low frequency power of heart rate spectrum.

## Data Availability

Not applicable.
